# Pharmacogenomics in epilepsy

**DOI:** 10.1016/j.neulet.2017.01.014

**Published:** 2018-02-22

**Authors:** Simona Balestrini, Sanjay M. Sisodiya

**Affiliations:** a NIHR University College London Hospitals Biomedical Research Centre, Department of Clinical and Experimental Epilepsy, UCL Institute of Neurology, London, and Epilepsy Society, Chalfont-St-Peter, Bucks, United Kingdom; b Neuroscience Department, Polytechnic University of Marche, Ancona, Italy

**Keywords:** Pharmacokinetics, Pharmacodynamics, Precision medicine, Epilepsy, Drug resistance, Drug response, Adverse drug reactions, Pharmacogenomics

## Abstract

There is high variability in the response to antiepileptic treatment across people with epilepsy. Genetic factors significantly contribute to such variability. Recent advances in the genetics and neurobiology of the epilepsies are establishing the basis for a new era in the treatment of epilepsy, focused on each individual and their specific epilepsy. Variation in response to antiepileptic drug treatment may arise from genetic variation in a range of gene categories, including genes affecting drug pharmacokinetics, and drug pharmacodynamics, but also genes held to actually cause the epilepsy itself.

From a purely pharmacogenetic perspective, there are few robust genetic findings with established evidence in epilepsy. Many findings are still controversial with anecdotal or less secure evidence and need further validation, e.g. variation in genes for transporter systems and antiepileptic drug targets. The increasing use of genetic sequencing and the results of large-scale collaborative projects may soon expand the established evidence.

Precision medicine treatments represent a growing area of interest, focussing on reversing or circumventing the pathophysiological effects of specific gene mutations. This could lead to a dramatic improvement of the effectiveness and safety of epilepsy treatments, by targeting the biological mechanisms responsible for epilepsy in each specific individual.

Whilst much has been written about epilepsy pharmacogenetics, there does now seem to be building momentum that promises to deliver results of use in clinic.

## Highlights

• Genetic variation can influence response to antiepileptic drug (AED) treatment through various effector processes.
• Metabolism of many AEDs is mediated by the cytochrome P450 (CYP) family; some of the CYPs have allelic variants that may affect serum AED concentrations.
• ‘Precision medicine’ focuses on the identification of an underlying genetic aetiology allowing personalised therapeutic choices.
• Certain human leukocyte antigen, *HLA*, alleles are associated with an increased risk of idiosyncratic adverse drug reactions.
• New results are emerging from large-scale multinational efforts, likely imminently to add knowledge of value from a pharmacogenetic perspective.

## 1 Introduction

The response, in terms both of seizure control and adverse reactions (ADRs), to antiepileptic drugs (AEDs) varies greatly across individuals [1]. Moreover, AED treatment in epilepsy is complicated because there are many different syndrome and seizure types within epilepsy, the biology of almost all of which is largely unknown. Response rates do seem to vary in relation to epilepsy syndrome, underlying cause, and other factors [2], [3]. The broad phenotypic spectrum and heterogeneous aetiology make the choice of treatment both challenging and empirical: evidence-based information guiding clinicians on the most effective drug and dose for individual patients is lacking. Furthermore, AEDs can have many associated ADRs, some of which are severe and life-threatening [4]. A cross-sectional survey of 809 patients showed that 36.5% experienced one or more ADRs; these events were not related to the number of AEDs, but rather to individual susceptibility, the specific AED used and physicians’ skills [5].

There is established evidence that genetic factors certainly contribute to this variability [6]. However, few robust findings have clearly emerged in epilepsy. Progress since the most recent comprehensive reviews [6], [7] has been limited. The increasing application of massively-parallel genetic sequencing and results of collaborative projects (such as EpiPGX, http://www.epipgx.eu/and CPNDS, http://cpnds.ubc.ca/) may soon expand knowledge in this area.

Recent advances in the genetics and neurobiology of the epilepsies are establishing the basis for a new era in the treatment of epilepsy, focused on each individual and their particular epilepsy. Testing for gene variations that might predict drug response and ADRs will hopefully soon improve the efficacy and safety of epilepsy therapies, targeting the best drug from those available for each individual patient. Increasing knowledge of the biology of the epilepsies may also lead to the re-purposing for epilepsy of drugs not originally intended for use in epilepsy, and may also direct discovery of rational new therapies. Moreover, as more is becoming understood, it is also clear that in some cases, there is important overlap between disease causation and the profile of response to AEDs.

There remains much to be learnt about epilepsy pharmacogenomics: any classification is necessarily arbitrary. Here, we distinguish the influence of genetic factors on response to AEDs from those affecting adverse drug reaction. We further classify the former according to the mediating mechanisms: pharmacokinetics and pharmacodynamics, or genes mutations which are recognised as capable of causing epilepsy (‘epilepsy genes’). This should be considered as an evolving classification. Despite ongoing progress in the field, only some findings are accepted within the community so far, whilst many results have not been replicated, and might be specific to certain populations. Below, we present the state of the art, by reporting first the findings with the best established evidence, followed by those with less certain status.

### 1.1 Influence of genetic factors on response to AEDs

Genetic variation can influence response to AEDs through various mediating effector systems, including pharmacokinetics and pharmacodynamics (e.g. polymorphism in gene encoding drug metabolizing enzymes or putative brain AED targets, such as receptors or ion channels), and mutations in ‘epilepsy genes’; and by modifying the expression of enzymes and other molecules involved in the pathogenesis of pharmacoresistance or adverse drug reactions [8], [9]. A key problem is that the mechanistic basis of pharmacoresistance, especially resistance to multiple AEDs, is not understood in the vast majority of cases; nor is the overlap between drug resistance and disease causation well understood [10].

### 1.2 Pharmacokinetics and pharmacodynamics

In humans, metabolism of those AEDs that undergo such processes is mostly mediated by the cytochrome P450 (CYP) family. Some of the CYPs have genetic (allelic) variants, encoding isoforms of differing activity, which in turn may affect serum AED concentrations, or alter flux through paths for drug metabolism, with subsequent potential risk of drug toxicity.

## 2 Established evidence

There is established evidence of an effect of polymorphic *CYP2C9* and *CYP2C19* genes: variant alleles can lead to significant differences in AED serum concentrations [11]. CYP2C9 accounts for about 90% of the metabolism of phenytoin. *CYP2C9* polymorphisms are an important determinant of the rate of phenytoin metabolism. Individuals carrying *CYP2C9* alleles encoding variant enzymes (allozymes) with reduced activity metabolize phenytoin at a considerably slower rate compared with individuals homozygous for the wild-type (CYP2C9*1; rs1057910(A)) allele, and therefore have a greater risk of developing concentration-dependent neurotoxicity: CYP2C9*2 (rs1799853) and CYP2C9*3 (rs1057910(C)) are the best documented [12], [13]. The maximum dose of phenytoin reported in a series of people with epilepsy was about 50 mg less per CYP2C9*3 allele [14].

A genome-wide association study of cases with phenytoin-related severe cutaneous adverse reactions and 412 population controls from Taiwan discovered a cluster of 16 single nucleotide polymorphisms in *CYP2C* genes at 10q23.33 that reached genome-wide significance. Direct sequencing of *CYP2C9* identified missense variant rs1057910 (CYP2C9*3) as showing significant association with phenytoin-related severe cutaneous adverse reactions [15]. The mechanism underlying this association has yet to be established. Despite the available evidence, pre-treatment pharmacogenetic testing for *CYP2C9* variants is not routine practice, with monitoring for clinical signs of toxicity and serum drug level being the standard approach.

## 3 Less robust evidence

Few, and mostly preliminary, data are available on genetic factors influencing the metabolism of other AEDs.

Studies investigated the association between *CYP2C19* genotypes and the pharmacokinetics of clobazam and N-desmethylclobazam (N-clobazam), a pharmacologically active metabolite that reaches higher serum concentrations than clobazam [16]. *CYP2C19* polymorphisms were associated with the serum concentration of N-clobazam and with clinical efficacy, thus indicating a gene-dose effect [17], [18], [19], [20], [21]. These were conducted mostly in Asian populations, and have not been replicated more widely.

Genetic influences on phenobarbital metabolism seem to relate mostly to *CYP2C19* polymorphism, with ethnic differences in the tolerability profile of phenobarbital [22]. However, there is absence of evidence that genotyping improves the outcome of phenobarbital therapy compared with clinical observation and serum drug concentration monitoring.

Valproate (VPA) is subject to complex oxidative and non-oxidative metabolic pathways. The majority of the drug is eliminated as glucuronide conjugates. Mitochondrial beta–oxidation, producing unsaturated metabolites, is the second major metabolic pathway. About 15–20% of the VPA dose is metabolized by CYP enzymes, resulting in the formation of 4–ene-VPA and hydroxy-metabolites [23]. The main enzyme for hydroxylation and desaturation to 4-ene-VPA is CYP2C9, with minor contributions from CYP2A6 and CYP2B6 [24].

Lamotrigine is eliminated almost entirely by glucuronidation. A previous pharmacokinetic study [25] found that in seven individuals with Gilbert’s syndrome (unconjugated hyperbilirubinaemia due to genetically-determined deficiency in uridine diphosphate glucuronosyltransferase, UGT), lamotrigine clearance was lower than in healthy controls. The pharmacogenetic impact of this in epilepsy is unknown.

Zonisamide is eliminated via renal excretion of the 2-sulfamoylacetyl-phenol (SMAP)-glucuronide (50%), native unchanged form (35%) and N-acetyl zonisamide (15%) [26]. In vitro data showed that the formation of SMAP is catalyzed mainly by CYP3A4 and to a minor extent by CYP3A5 and CYP2C19 [27]. Okada et al. [28] found that *CYP2C19* genotypes may influence the pharmacokinetics of zonisamide, with a role in the development of some adverse reactions, in Japanese patients with epilepsy. Again, these findings need replication.

Carbamazepine is extensively metabolized in the liver, with less than 5% of an oral dose excreted unchanged in urine [29]. Carbamazepine is predominantly metabolized to carbamazepine-10,11-epoxide by CYP3A enzymes. The main enzyme involved in its metabolism, CYP3A4, has a high number of known polymorphisms although most have a very low frequency and do not reflect significant inter-individual variability in the phenotypic effect in vivo [30]. Carbamazepine-10,11-epoxide and carbamazepine- 10,11-*trans*-diol are the primary (≤ 60%) metabolites in urine [31]. Many CYP enzymes are involved in the formation of carbamazepine metabolites [32] but no significant contribution of *CYP* genotypes to the pharmacokinetics of carbamazepine has been demonstrated so far. Single nucleotide polymorphisms of the microsomal epoxide hydrolase (*EPHX1*) gene have been shown to affect carbamazepine pharmacokinetics in Chinese people with epilepsy [33], and in Kosovan people with epilepsy of Albanian ethnicity [34].

A further series of reports are all in need of replication. Retigabine is metabolized by extensive N-glucuronidation and N-acetylation. However, its clearance seems not to be affected in Gilbert’s syndrome nor by common N-acetyl-transferase type 2 (NAT-2) polymorphisms [35]. A study in Han Chinese people with epilepsy found an association between the effects of *SCN1A*, *ABCC2* and *UGT2B7* genetic polymorphisms and oxcarbazepine maintenance doses [36]. Genetic contribution of *CYP1A1* alleles on treatment outcome in people with epilepsy was studied in an Indian population. In particular, the variant rs2606345 (resulting in reduced CYP1A1 expression) was associated with poor response to first-line AEDs in Indian women with epilepsy [37], [38].

## 4 Unclear evidence

Drug transporters are involved in the protection of cells and organs through active extrusion of xenotoxins, including many drugs. Several ATP-dependent transport proteins have been associated with drug resistance. One of the best studied transporters is P-glycoprotein (P-gp), encoded by the ATP-binding cassette sub-family B member 1 gene, *ABCB1* [39]. In the brain, P-gp is expressed in astrocytes, endothelial cells and neurons, and there is evidence that its overexpression in epileptogenic tissue can contribute to AED resistance [40]. Several studies have demonstrated a link between *ABCB1* gene variants and the response to treatment in epilepsy, but most studies have in fact been inconclusive [41], [42], [43], [44]. A recent meta-analysis including 8,604 subjects from 30 studies identified a significant correlation between the *ABCB1* C3435T polymorphism and drug-resistant epilepsy, a result that needs to be verified in a case-control study with a larger sample size, as acknowledged by the authors [45]. Evidence for an association of *ABCB1* polymorphisms with AED resistance is still unclear and does not justify routine testing to predict response to AED therapy.

There have been associations reported for variation in other transporter-encoding genes and treatment-resistant epilepsy [36], [46], [47], [48], but the strength of evidence is less still than that for *ABCB1*.

Research into polymorphisms affecting genes encoding AED targets, such as voltage-gated ion channels [49], [50], the GABA-A receptor [51], or synaptic vesicle proteins [52], has not found any significant association with drug response [53]. More data are likely to emerge in this area as large consortia sequencing large numbers of people with epilepsy report their findings.

### 4.1 Epilepsy genes

‘Precision medicine’ is an approach for disease treatment and prevention based on individual variability in genes, environment, and lifestyle for each person [54]. This approach, incorporating the identification of an underlying genetic aetiology to allow personalised therapeutic choice, or to drive re-purposing of drugs, builds upon thoughtful clinical practice that has been applied for years. The ‘precision medicine’ paradigm has become more realistic with advances in discovery of more of the putative causes of epilepsy in individual patients, and with the wider availability of the necessary genetic technologies [9], [55], [56]. Formal quantitative evaluation of environmental variables is still largely unaddressed, whilst lifestyle issues have always featured in any holistic approach to the delivery of care.

In epilepsy, if a specific gene mutation causes a functional alteration of physiological systems involved in the control of brain excitability, a rational treatment strategy might ideally aim to reverse or circumvent the dysfunction. This approach may not always prove successful, however, for a number of reasons, including the fact that no causal variant acts in isolation, but does so in the context of the rest of the genome and its variation, and because compensatory and adaptive changes may become fixed and difficult, or impossible, to reverse with treatment of the perceived original fault. The current targeted treatment approach in precision medicine requires the identification of the underlying causative genetic alteration, determination of the functional alteration of the physiological system caused by the genetic mutation, and evaluation of the effect of treatment putatively intended and able to reverse or inhibit the functional alteration.

The current evidence is ordered by the robustness of the available evidence.

## 5 Established evidence

An established example of the application of the precision medicine concept is in the management of GLUT-1 deficiency, a genetic metabolic encephalopathy due to mutations in the *SLC2A1* gene, which encodes the glucose type I transporter (GLUT-1), resulting in impaired transport of glucose across the blood-brain barrier. GLUT-1 deficiency shows wide phenotypic pleiotropy, including intellectual disability, movement disorder, and drug-resistant epilepsy, as AEDs usually fail to control seizures. The gold standard treatment is the ketogenic diet, which provides ketones as an alternative fuel for cerebral metabolism, thereby treating the symptoms of neuroglycopenia [57]. Early diagnosis and initiation of ketogenic diet are crucial to provide brain nourishment and control seizures [58], although the benefit on neurodevelopment seems controversial [59]. The challenge is to think of GLUT-1 deficiency as a possible diagnosis, an issue shared by many genetic diagnoses in epilepsy because of the pleiotropic manifestations of many mutated genes: the use of gene panels or more extensive sequencing (exome or genome) can help overcome this difficulty.

Pyridoxine (vitamin B6)-dependent epilepsy is caused by bi-allelic mutations in the *ALDH7A1* gene, which encodes antiquitin. Deficiency of antiquitin causes seizures because accumulating Δ^1^-piperideine-6-carboxylate (P6C) condenses with pyridoxal 5′-phosphate (PLP) and inactivates this enzyme cofactor, which is essential for normal metabolism of neurotransmitters. *ALDH7A1* analysis could also be used for prenatal diagnosis of pyridoxine-dependent epilepsy and seizures are often fully controlled by treatment with pyridoxine [60]. B6-responsive seizures may also be due to mutations in pyridox(am)ine 5'-phosphate oxidase (*PNPO*) gene, and in some cases may be better treated with pyridoxal 5′-phosphate [61].

## 6 Less robust evidence

Other recent reports hint at the potential relevance of precision medicine in epilepsy, by investigating the effect of treatment of the molecular defects resulting from specific mutation in certain genes. There are few examples so far and most are anecdotal.

Dravet Syndrome is a severe epilepsy syndrome of early childhood, associated with drug resistance, developmental slowing or regression and intellectual disability, and risk of premature mortality, including sudden unexpected death in epilepsy (SUDEP). It is perhaps the best understood genetic epilepsy and has advanced understanding not only of concepts in disease biology, but also of treatment biology and genetics. The most frequent cause of Dravet Syndrome is mutation in the voltage-gated sodium channel alpha1 subunit gene (*SCN1A*). Mutations in *SCN1A* are also associated with milder phenotypes, such as genetic epilepsy with febrile seizures plus (GEFS + ) [62]. Though of all the epilepsies (especially the non-metabolic ones), we understand Dravet Syndrome the best, its pathophysiology has not been fully explained yet and this makes the development of targeted treatment more complicated. The “interneurone hypothesis” is currently the best-supported pathophysiological explanation of Dravet Syndrome. According to this hypothesis, *SCN1A* mutation results in reduced function of GABAergic inhibitory interneurons, leading to an overall excessive neuronal excitation [63], [64], [65]. However, other studies using Dravet Syndrome patient-derived induced pluripotent stem cells (iPSC) found increased excitability of both GABAergic and glutamatergic isolated neurons [66]. A recent study identified a depolarizing GABA phenomenon, and explored the mechanism of action of benzodiazepines and stiripentol, commonly beneficial in Dravet Syndrome, by using a physiology-based computational model [67]. It is still unclear if major manifestations of the disease are caused by disturbances in embryonic development or by persistent SCN1A deficiency in later life. It is also not known if increasing SCN1A expression after birth would alter the disease phenotype. A recent study demonstrated a new regulatory mechanism of *SCN1A* expression, by targeting a long non-coding RNA, both in vitro and in vivo, in the brain of Dravet knock-in mouse model and a non-human primate (African green monkey model); upregulation of haploinsufficient SCN1A expression led to significant improvements in seizure phenotype and excitability of hippocampal interneurons [68]. As a paradigmatic epileptic encephalopathy, Dravet Syndrome still has much to teach us.

Currently, treatment of Dravet Syndrome typically involves polytherapy with valproate, clobazam and often stiripentol. Evidence for many drugs favoured in Dravet Syndrome is in fact sparse [69]. Stiripentol is the only compound to have efficacy in Dravet Syndrome formally demonstrated in a randomised controlled trial (when combined with valproate and clobazam; [70]); however, the use of stiripentol, valproate and clobazam does not always yield complete seizure freedom and may cause adverse side effects [71], [72]. New and effective treatment strategies with possibly novel mechanisms are therefore needed.

Many model systems have been employed to explore the cellular mechanisms of seizure genesis caused by different *SCN1A* mutations [73]. Heterologous expression systems, in which cloned sodium channel α-subunits are expressed in an intrinsically non-excitable cell, were the first models used to investigate the cellular consequences of *SCN1A* mutations associated with epilepsy. Studies in heterologous systems were the first to demonstrate that *SCN1A* mutations can cause both gain and loss of function at the channel level [74]. There is now growing evidence that epileptogenic *SCN1A* mutations cause mainly loss of function, whereas gain of function is found for mutations that cause familial hemiplegic migraine [75]. Heterologous expression systems have been used for testing compounds that modulate voltage-gated sodium channels. For example, ranolazine, a U.S. Food and Drug Administration (FDA)-approved drug for chronic angina treatment, was first identified as a selective blocker of persistent currents in mutant Na_V_1.1 channels expressed in one of these models [76]. In follow-up studies in cultured rat hippocampal neurons, ranolazine was found to reduce neuronal excitability and suppress epileptiform activity evoked by NMDA receptor activation [77]. A more recent study showed that ranolazine does not inhibit the persistent Na+ current more strongly than phenytoin in central neurons, but is a better use-dependent blocker of transient Na+ current than phenytoin [78]. Furthermore, identifying compounds that can differentially modulate different subtypes/isoforms of sodium channels may contribute to identifying new treatment strategies for *SCN1A*-related epilepsy [79]. Mouse models have shown how the same *SCN1A* mutation can have differential effects on different types of neurons, and how the same type of mutation, such as *SCN1A* truncations, may have different effects on neuronal excitability depending on developmental stage [65], [80].

Baraban et al. [81] characterised zebrafish Na_v_1.1 (*scn1Lab*) mutants originally identified in a chemical mutagenesis screen using the optokinetic response as an assay [82]. The zebrafish *scn1Lab* gene shares a 77% identity with human *SCN1A* and is expressed in the central nervous system. Baraban et al. [81] demonstrated that mutants exhibit hyperactivity, including convulsive behaviour, spontaneous electrographic seizures, shortened lifespan and a pharmacological profile similar to the human condition. They then used the validated model in a novel high-throughput screening program to identify compounds that ameliorated the epilepsy phenotype. The strategy identified clemizole, a US Food and Drug Administration (FDA)-approved compound but not a licensed AED, as an effective inhibitor of spontaneous convulsive behaviour and electrographic seizures in these mutants. There have been no further reports on clemizole use in Dravet Syndrome or models. Recent data suggest that fenfluramine may be effective in Dravet syndrome [83], [84]. This drug was initially developed as an appetite suppressant, but withdrawn from the market due to serious adverse effects, including valvular heart disease and pulmonary hypertension [85], [86]. Fenfluramine has serotoninergic effects [87], but the exact anti-seizure mechanism has not been elucidated yet. Fenfluramine significantly reduced epileptiform discharges in *scn1Lab* morphants in recent studies [88], [89]. Currently there are four ongoing clinical trials to evaluate the effectiveness and tolerability of this drug in Dravet Syndrome. A functional link between the serotonergic and GABAergic pathway has recently been elucidated since 5-HT2A agonism elevates the activity of GABAergic interneurons [90]. A recent study in zebrafish suggested a dual mechanism in Dravet Syndrome pathophysiology, postulating that GABA interneurons are defective, not only due to presence of mutations but also by a relative deficiency in locally released serotonin [91]. If confirmed, this supports serotonergic receptor modulation as a promising therapeutic target in Dravet Syndrome.

Some patients with Dravet Syndrome have seizure aggravation with exposure to sodium channel blockers, e.g. carbamazepine, lamotrigine and phenytoin [92]. As Na_v_1.1 shows higher expression in inhibitory bipolar as compared to excitatory pyramidal neurons [93], it can be hypothesized that the effect of sodium channel blockers on already impeded inhibitory interneurons outweighs the effect on excitatory neurons leading to a further decrease in inhibition and seizure exacerbation. However, it has been shown how lamotrigine can improve seizure control in some patients with Dravet Syndrome [94]. All these findings show how treatment response maybe specific to the nature and the location of the *SCN1A* mutation [75], [95], and offers up a somewhat daunting perspective of precision medicine in the epilepsies.

The NMDA receptor is a ligand-gated ion channel activated by glutamate. *GRIN2A* encodes a subunit of the NMDA receptor. Mutations and deletions in the *GRIN2A* gene predispose to several childhood-onset epilepsy syndromes within the epilepsy-aphasia spectrum, including Landau-Kleffner syndrome (LKS), epileptic encephalopathy with continuous spike-and-wave during sleep (ECSWS), childhood epilepsy with centrotemporal spikes (CECTS), atypical childhood epilepsy with centrotemporal spikes (ACECTS), autosomal dominant rolandic epilepsy with speech dyspraxia (ADRESD), and infantile-onset epileptic encephalopathy [96], [97], [98], [99], [100]. Under normal conditions of synaptic transmission, very few ions pass through the channel because magnesium (Mg^2+)^ blocks the pore. Upon receptor activation, Mg^2+^ is displaced, which allows calcium (Ca^2+)^ and other cations to move into the cell. A dysfunctional NMDA receptor can allow excess calcium influx into the cell. In vitro experiments testing activity of a *GRIN2A* mutant (L812 M) receptor showed increased activity in response to agonists and decreased response to negative modulators [101]. In vitro analysis showed that memantine, a *N*-methyl-d-aspartate (NMDA) receptor antagonist, inhibited the increased activity of the NMDA receptor caused by the L812 M mutation. A child with an early onset epileptic encephalopathy due to this *GRIN2A* missense mutation (L812 M) [102] was treated with memantine added on to his antiepileptic medication regimen, and had a significant decrease in seizure frequency and improvement in interictal EEG recordings. In contrast, another child’s epileptic encephalopathy was found to be caused by a different *GRIN2A* missense mutation (N615 K) causing a different channel dysfunction, with no effect on receptor activity, but acting through the relief of Mg^2+^ blockade [96]. Treatment with memantine was therefore not tried in this case, as it was not indicated.

A child with a different epilepsy syndrome, migrating partial seizures of infancy (an early onset epileptic encephalopathy syndrome which is typically drug-resistant), due to a gain of function mutation in the gene *KCNT1*, was treated with the antiarrhythmic drug quinidine. This drug is a partial antagonist of KCNT1, and the treatment was associated with a marked reduction in seizure frequency and improved psychomotor development [103]. Quinidine was previously used to reverse the hyperactivity of the mutant *KCNT1* in Xenopus oocytes [104]. A second case with *KCNT1*-related epilepsy, showing a novel phenotype with developmental regression and severe nocturnal focal and secondarily generalised seizures starting in early childhood, did not respond to treatment with quinidine [105]. Currently the therapeutic effects of quinidine in *KCNT1*-related epilepsy remain largely unknown and more work is required, although it seems a promising treatment option due to gain-of-function mutations in *KCNT1*.

*KCNQ2*-related epilepsy is another example where a molecular diagnosis may influence the therapeutic choice. Retigabine (ezogabine), is a drug primarily acting as a positive allosteric modulator of KCNQ2-5 (K_v_7.2–7.5) ion channels: it is the first neuronal potassium (K + ) channel opener licensed for the treatment of epilepsy [106]. In vitro studies identified the probable binding site of retigabine in KCNQ2 and KCNQ3 channels, explaining its voltage-dependent activating effect through a hyperpolarizing shift of the activation curve [107], [108]. *KCNQ2* and *KCNQ3* mutations have been associated with a phenotypic spectrum ranging from a benign form of neonatal epilepsy (BFNS) [109] to a severe form of early-onset epileptic encephalopathy [110], [111]. Orhan et al. [112] defined the disease mechanism of seven de novo missense *KCNQ2* mutations associated with severe epileptic encephalopathy and found a clear loss of function for all the mutations studied in vitro. Most mutations showed a dominant-negative effect on wild-type KCNQ2 or KCNQ3 subunits. The use of retigabine partially reversed the loss of function, in vitro, for the majority of analyzed mutations. Preliminary data from humans with *KCNQ2*-related disease suggest retigabine may be a useful treatment option [113], but more data are required. Sodium channel blockers also seem effective in *KCNQ2*-related epilepsy [111], [114], possibly because voltage-gated sodium channels and KCNQ potassium channels co-localize and are bound at critical locations of the neuronal membrane [115]; modulation of one channel may significantly affect the function of the channel complex and this may explain the efficacy of sodium-channel blockers that have a modulating effect on both channels [111]. Sodium channel blockers including carbamazepine and phenytoin should also be considered as first-line treatment in patients with *KCNQ2*-related epilepsy [116].

*SCN2A* encodes the alpha2 subunit of the neuronal sodium channel and has also been associated with a wide phenotypic spectrum of epilepsy syndromes ranging from benign familial neonatal-infantile seizures to epilepsy of infancy with migrating focal seizures or other severe epileptic encephalopathies [117], [118], [119]. Sodium channel blockers have shown significant effectiveness in *SCN2A*-epileptic encephalopathies. The mechanism of the effect has not been elucidated yet, but, as might be expected, the effect seems mostly present in patients with gain-of-function mutations [118], [119]. An anecdotal case with a de novo *SCN2A* splice site mutation associated with epileptic encephalopathy, early onset global developmental delay, intermittent ataxia, autism, hypotonia, and cerebral/cerebellar atrophy was recently reported. In the cerebrospinal fluid, both homovanillic acid and 5-hydroxyindoleacetic acid were significantly decreased; extensive biochemical and genetic investigations ruled out primary neurotransmitter deficiencies and other known inborn errors of metabolism. Treatment with oral 5-hydroxytryptophan, l-Dopa/Carbidopa, and a dopa agonist resulted in mild improvement of seizure control, most likely via dopamine and serotonin receptor activated signal transduction and modulation of glutamatergic, GABA-ergic and glycinergic neurotransmission [120]. This single case hints at the complexity that is likely to be observed when additional or compensatory changes are present in ‘monogenic’ epilepsies.

*SCN8A* encodes the voltage-dependent sodium channel Na_v_1.6, located in both inhibitory and excitatory neurons [121]. Mutations in this gene have been found in 0.6-2.4% of cases with early infantile epileptic encephalopathy [122], [123] and have been associated with increased risk of SUDEP [121], [123], [124], [125]. In most cases, *SCN8A* mutations occurred de novo and showed a gain-of-function effect [121], [126]. There is clinical evidence of a possible effective precision medicine approach in using sodium channel blockers to treat patients with *SCN8A*-related epilepsy [121], [123], [127], in particular if the mutations are known to cause gain of function [128].

Tuberous sclerosis complex (TSC) is an autosomal dominant, multi-organ disease with widely variable clinical expression. Approximately 85% of patients with TSC are found to have a mutation in one of two genes, *TSC1*, encoding hamartin, or *TSC2*, encoding tuberin [129]. Protein products of these genes have been shown to form a heterodimer (TSC1–TSC2 complex) that inhibits the mechanistic target of rapamycin (mTOR) signaling cascade [130]. The mTOR signaling cascade regulates processes involved in cell growth and homeostasis in response to many metabolic cues. Dysregulated, inadequate suppression of the mTOR pathway results in dysplastic lesions in multiple organ systems, including cortical tubers, radial glial bands, subependymal nodules, and subependymal giant cell tumor formation in the fetal and developing brain [131]. Epilepsy is the most common neurologic symptom in patients with TSC [132]. Rapamycin (otherwise known as sirolimus) is an inhibitor of mTOR. Rapamycin prevents activation of T cells and B cells by inhibiting their response to interleukin-2 (IL-2), and is an FDA-approved drug for immunosuppression after organ transplantation. A clinical trial of rapamycin for renal angiomyolipomata associated with tuberous sclerosis or lymphangioleiomyomatosis revealed an almost 50% decrease in angiomyolipoma volumes by the end of the 12-month rapamycin administration period [133]. A phase 2 multicenter trial was conducted to evaluate the efficacy and tolerability of sirolimus for the treatment of kidney angiomyolipomata [134]. Treatment with sirolimus for 52 weeks induced regression of kidney angiomyolipomata, subependymal giant cell astrocytomas, and liver angiomyolipomata. Another clinical trial was designed to study the impact of everolimus (a derivative of sirolimus) on subependymal giant cell astrocytoma growth and showed sustained effect on tumor reduction over ≥5 years of treatment, with no safety concerns [135], [136]. Treatment with rapamycin was shown to prevent the development of epilepsy and premature death in mouse models of TSC [137], [138]. A few reports in humans have showed significant improvement in seizure control in children and adults with TSC-associated epilepsy, with a tolerable safety profile [139], [140], [141]. More studies are needed, and are important as evidence grows for a more widespread dysregulation of the mTOR pathway beyond that seen in TSC alone. One example comes from epilepsies due to mutation in a gene in this pathway.

*DEPDC5* (Dishevelled, Egl-10 and Pleckstrin Domain Containing Protein 5) loss-of-function mutations have recently been reported in a variety of genetic focal epilepsy syndromes, including familial focal epilepsy with variable foci, autosomal dominant nocturnal frontal lobe epilepsy, familial temporal lobe epilepsy [142], [143], [144], rolandic epilepsy and other non-lesional focal childhood epilepsies [145] and focal epilepsy associated with focal cortical dysplasia, both familial and sporadic [146], [147]. *DEPDC5* variants have also been found in cases with malformation of cortical development [146], [147] and in one sporadic case with focal epilepsy among a cohort of French-Canadian individuals [148]. *DEPDC5* encodes a protein that is expressed ubiquitously in developing and adult brain [142]; it has GTPase-activating protein (GAP) activity and is part of the GATOR1 complex, a negative regulator of the mechanistic target of rapamycin complex 1 (mTORC1), in the mTOR pathway [149]. The mTORC1 pathway is dysregulated in several neurological disorders associated with cortical malformations and drug-resistant epilepsy, such as tuberous sclerosis [150] and hemimegalencephaly [151]. Because *DEPDC5* acts as a repressor of mTOR activity, *DEPDC5* mutations are predicted to result in upregulation of the mTOR signaling pathway. Prenatal treatment with rapamycin in global *Depdc5* knockout rats rescued growth delay and embryonic lethality, and prevented enhanced cell size and dysmorphism of neurons [152]. Disruption of the mTOR signaling pathway is a putative pathophysiologic mechanism of epileptogenesis in different models of lesional epilepsy in rodents and humans [153] and has emerged as a possible therapeutic target for epilepsy [154]. Deep whole-exome sequencing identified mutations of the *MTOR* gene in 12 of 77 subjects with focal cortical dysplasia type II (FCDII), a sporadic developmental malformation of the cerebral cortex characterised by dysmorphic neurons, dyslamination and medically-refractory epilepsy [155]. The identified mutations induced the hyperactivation of mTOR kinase. Focal cortical expression of mutant mTOR by in utero electroporation in mice was sufficient to disrupt neuronal migration and cause spontaneous seizures and cytomegalic neurons. Inhibition of mTOR with rapamycin suppressed cytomegalic neurons and epileptic seizures, so identifying mTOR as a treatment target for intractable epilepsy in focal cortical dysplasia [155]. Germline heterozygous mutations in the genes encoding the two other components of GATOR1, *NPRL2* (nitrogen permease regulator-like 2), and *NPRL3* (nitrogen permease regulator-like 3), have been reported. An *NPRL3* pathogenic mutation was discovered by whole exome sequencing in a family including four subjects with focal epilepsy, two of whom had focal cortical dysplasia type IIa, and later identified in two additional unrelated patients with focal cortical dysplasia type IIa [156]. A second study using whole exome sequencing in a large family with autosomal dominant nocturnal epilepsy without apparent structural brain abnormalities uncovered an inactivating variant in *NPRL2* [157]. Two other studies identified several *NPRL2* and *NPRL3* mutations in both sporadic and familial focal epilepsies using targeted gene panel sequencing [158], [159]. GATOR1 complex gene mutations leading to mTORC1 pathway upregulation are an important cause of focal epilepsy with cortical malformations. Treatment with mTOR inhibitors in patients with gene mutations affecting the GATOR1 complex subunit has not yet been investigated to our knowledge. However pathophysiological similarities between different mTORopathies warrant further studies [146].

PRICKLE proteins, such as PRICKLE1, are core constituents of the planar cell polarity signaling pathway that establishes cell polarity during embryonic development [160]. Mutations in the *PRICKLE* genes can cause seizures in humans, zebrafish, mice, and flies, suggesting a seizure-suppression pathway that may be evolutionarily conserved [161], [162]. This pathway has never been targeted for novel anti-seizure treatments. Paemka et al. [163] defined the mammalian PRICKLE-interactome, identifying PRICKLE-interacting proteins that localize to synapses and a novel interacting partner, USP9X, a substrate-specific de-ubiquitinase. Their findings demonstrated that inhibition of USP9X can arrest PRICKLE-mediated seizures. As *PRICKLE* mutant humans, mice, zebrafish, and Drosophila all exhibit seizures [162], [164], USP9X-modulating molecules may be worth pursuing as a new class of anti-seizure therapy. This represents an example of the translational power of studying diseases in species across the evolutionary spectrum.

Based on all these reports, the ascertainment of a molecular diagnosis early in life and generation and characterization of cell culture and valid animal models for the interpretation of the functional consequences of a mutation, may become fundamental for overall diagnosis, prognosis and therapeutic management. Currently, genetically-targeted therapies are available for only a minority of genetic epilepsies. Such therapies include drugs that exert their effect on ion channels, inhibit the mTOR pathway or provide alternative energy supply to the brain by avoiding defective transport mechanisms. So far, drugs or treatment that have been investigated were those already available on the market but not necessarily approved for the treatment of epilepsy. Novel drug generation has yet to come.

### 6.1 Influence of genetic factors on adverse drug reactions

Genetic polymorphisms have been associated with the risk of side effects. In particular, there is extensive evidence of the association between certain human leukocyte antigen (*HLA*) alleles and increased risk of idiosyncratic adverse drug reactions.

## 7 Established evidence

A strong association was found in people with epilepsy and Han Chinese origin between a genetic marker, HLA-B*15:02, and Stevens-Johnson syndrome induced by treatment with carbamazepine, probably due the role of this allele in mediating the activation of cytotoxic T-lymphocytes [165], [166]. The frequency of the HLA-B*15:02 allele is very low in Caucasian people, but high in Han Chinese and other South Asian ethnic groups, including people from Thailand, Malaysia, Vietnam and other South Asian countries: the risk of HLA-B*15:02-associated Stevens-Johnson syndrome and toxic epidermal necrolysis is dependent on the specific population. A study conducted in Taiwan showed that screening patients for the HLA-B*15:02 allele before the initiation of carbamazepine treatment and withholding carbamazepine from HLA-B*15:02–carrying patients can reduce the incidence of carbamazepine-induced Stevens-Johnson syndrome and toxic epidermal necrolysis among patients of Han Chinese origin [167]. Regulatory agencies and therapeutic guidelines recommend that patients from Han Chinese and other South Asian ethnic groups be genotyped for HLA-B*15:02 before commencing treatment with carbamazepine, and that carbamazepine be avoided if at all possible in carriers of this allele [168], [169]. The HLA-B*15:02 allele has also been associated with increased risk of Stevens-Johnson syndrome/toxic epidermal necrolysis after treatment with other aromatic AEDs, including phenytoin [170], oxcarbazepine [171], and lamotrigine [170]. This is the best example of a useful pharmacogenetic variant in epilepsy.

HLA-A*31:01 has been associated with increased risk of carbamazepine-induced hypersensitivity reactions, ranging from maculopapular exanthema to severe blistering reactions, among subjects of European ancestry and in the Japanese population [172], [173]. Recent economic modelling suggested that routine testing for HLA-A*31:01 in order to reduce the incidence of cutaneous adverse drug reactions in patients being prescribed carbamazepine for epilepsy is likely to represent a cost-effective use of health care resources [174].

## 8 Unclear evidence

In some patients treated with VPA, severe hyperammonemia has been observed in the absence of liver failure and may result in encephalopathy with the following symptoms: vomiting, ataxia, behavioural changes, lethargy, somnolence, or, in extreme cases, coma [175]. The exact mechanism of VPA-induced encephalopathy has not been fully defined yet. A prospective multi-center study evaluated the putative association between the T1405 polymorphism in the carbamoyl phosphate synthetase 1 (*CPS1*) gene and occurrence of VPA-induced hyperammonemia in a cohort of 142 adult Caucasian patients with epilepsy treated with valproic acid for at least one year and with normal liver function. They found that this variant was a significant risk factor for the occurrence of hyperammonemia, even in patients with serum VPA levels within a specified therapeutic range [176]. Non-alcoholic fatty liver disease is another adverse reaction that can be induced by VPA treatment [177] and this chronic liver condition is associated with elevated serum levels of γ-glutamyltransferase (γ-GT) [178]. A retrospective study in Japanese patients showed that the Val16Ala polymorphism of the Superoxide Dismutase 2 (*SOD2*) gene, encoding an antioxidant enzyme with a critical role in the detoxification of mitochondrial reactive oxygen species, has an impact on the relationship between VPA exposure and γ-GT elevation. This was determined by using a population pharmacokinetic-pharmacodynamic modelling approach [179]. Weight gain, a common adverse reaction of VPA, has been associated with leptin receptor (*LEPR*) and ankyrin repeat kinase domain containing 1 (*ANKK1*) gene polymorphisms in a cohort of Han Chinese people with epilepsy [180]. All these findings need replication.

Although the mechanism of VPA-induced adverse effects is not clearly understood, there is evidence that its metabolic pattern is associated with the pathogenesis of adverse drug reactions. VPA-induced liver damage has been associated with CYP2C9-and CYP2A6-mediated formation of the hepatotoxic 4-ene metabolite [181]. In a paediatric cohort, Budi et al. [182] found that *CYP2C9* variant-guided treatment significantly reduced valproic acid misdosing. In the group of children (n = 52) who received a *CYP2C9*-status adapted dose, signs of toxicity, notably hyperammonemia, were reduced. These findings, as many others in epilepsy pharmacogenetics, still require replication.

There are other studies of genetic factors associated with adverse drug reactions, with unclear evidence. We made an arbitrary selection of some of the most recent or potentially clinically relevant studies. Additional studies can be found listed on the Epilepsy Genetic Association Database (epiGAD; http://www.epigad.org/).

A summary of all the findings presented is reported in Table 1.

**Table 1:** Influence of genetic factors on response and adverse reactions to AEDs through various mediators: summary of existing findings.

Response		
Mediator	Genetic factor	Effect [references]
*Pharmacokinetics and pharmacodynamics*	Variation in *CYP2C9* gene	Risk of developing concentration-dependent neurotoxicity from phenytoin [12], [13]- established evidence
	Variation in *CYP2C19* gene	Association with the serum concentration of N-desmethylclobazam and with its clinical efficacy, indicating a gene-dose effect [17], [18], [19], [20], [21]
	Variation in *CYP2C19* gene	Ethnic differences in the tolerability profile of phenobarbital [22]
	*UGT1A1* variants	Altered clearance of lamotrigine [25]
	Variation in *CYP2C19* gene	Risk of adverse reactions from zonisamide [28]
	Variation in *SCN1A, ABCC2, UGT2B7* genes	Association with oxcarbazepine maintenance doses [36]
	Variation in *CYP1A1* gene	Association with response to first-line antiepileptic drugs in Indian women [37], [38]
	*ABCB1* gene (encoding P-glycoprotein, P-gp, multidrug transporter) variants	Drug-resistant epilepsy [40], [41], [42], [43], [44], [45]
	Variation in genes coding for AED targets	No significant association with drug response [49], [50], [51], [52], [53]
*Epilepsy genes*	Mutations in the *SLC2A1* gene causing GLUT-1 deficiency syndrome	Gold standard treatment is the ketogenic diet, treating the symptoms of neuroglycopenia [[57], [58], [59]- established evidence
	Bi-allelic mutations in the *ALDH7A1* gene causing pyridoxine (vitamin B6)-dependent epilepsy	Gold standard treatment is pyridoxine or pyridoxal 5′-phosphate supplementation [60], [61]- established evidence
	*SCN1A*-related epilepsies	Stiripentol is effective in Dravet Syndrome (especially when combined with valproate and clobazam), established in a randomised controlled trial; however, the use of stiripentol, valproate and clobazam does not always yield complete seizure freedom and may cause adverse side effects [70], [71], [72]
		Ranolazine was identified as a selective blocker of persistent currents in mutant NaV1.1 channels expressed in heterologous expression systems [76], [77], [78]- uncertain evidence, recently it was shown that ranolazine does not inhibit the persistent Na+ current more strongly than phenytoin in central neurons
		Clemizole was identified as an effective inhibitor of spontaneous convulsive behaviour and electrographic seizures in zebrafish Na_v_1.1 (*scn1Lab*) mutants [81]
		Fenfluramine has serotoninergic effects and was shown to significantly reduce epileptiform discharges in *scn1Lab* morphants. Evidence of effectiveness in control of convulsive seizures and good tolerability in a small cohort of patients with Dravet Syndrome [83], [84], [85], [86], [87], [88], [89]- ongoing clinical trials in Dravet syndrome
		Use of sodium channel blockers can aggravate seizures in Dravet syndrome. Note, however, that lamotrigine can improve seizure control in some patients with Dravet Syndrome [92], [94]
	*GRIN2A* causing early-onset epileptic encephalopathy	Use of memantine, a *N*-methyl-d-aspartate (NMDA) receptor antagonist, can inhibit the increased activity of the NMDA receptor caused by specific mutations [102]
	*KCNT1-*related epilepsy	Use of quinidine, partial antagonist of KCNT1 used as antiarrhythmic drug, was shown to reduce seizure frequency and improve psychomotor development in one patient with migrating partial seizures of infancy [103] and was shown to reverse the hyperactivity of mutant KCNT1 in Xenopus oocytes [104]
	*KCNQ2*-related epilepsies	Use of retigabine (ezogabine), a positive allosteric modulator of KCNQ2-5 (K_v_7.2–7.5) ion channels, was shown to partially reverse the loss of function, in vitro, of missense *KCNQ2* mutations associated with severe epileptic encephalopathy [107], [108], [113]- only preliminary data in humans
		Sodium channel blockers seem effective in *KCNQ2*-related epilepsy, possibly because voltage-gated sodium channels and KCNQ potassium channels co-localize and are bound at critical locations of the neuronal membrane [111], [114], [115], [116]
	*SCN2A*-related epilepsies	Sodium channel blockers have shown significant effectiveness in *SCN2A*-epileptic encephalopathies; pathophysiological considerations suggest that sodium channel blockers should only be applied in patients with gain-of-function mutations [118], [119]
		Treatment with oral 5-hydroxytryptophan, l-Dopa/Carbidopa, and a dopa agonist resulted in mild improvement of seizure control, in a case with a de novo *SCN2A* splice site mutation associated with epileptic encephalopathy [120]
	*SCN8A-*related epilepsies	Sodium channel blockers have shown effectiveness in patients with gain-of-function mutations; in rare cases an apparent loss-of-function effect has been described but it remains unclear if these may show gain-of-function effect in vivo [121], [123], [127], [128]
	Tuberous sclerosis complex (TSC) associated with *TSC1* or *TSC2* mutations	Rapamycin (sirolimus), an inhibitor of the mechanistic target of rapamycin (mTOR) signaling cascade, was shown to prevent the development of epilepsy and premature death in mouse models of TSC and to significantly improve seizure control in children and adults with TSC-associated epilepsy, with a tolerable safety profile [137], [138], [139], [140], [141]
	*DEPDC5*-related epilepsies	*DEPDC5* encodes a protein that is part of the GATOR1 complex, a negative regulator of the mechanistic target of rapamycin complex 1 (mTORC1), in the mTOR pathway; its mutations are predicted to result in upregulation of the mTOR signaling pathway. Inhibition of mTOR pathway with rapamycin was shown to suppress cytomegalic neurons and epileptic seizures in model systems [152], [153], [154]. No studies on treatment with mTOR inhibitors in patients with gene mutations affecting the GATOR1 complex subunit
	*PRICKLE*-mediated seizures	USP9X, a substrate-specific de-ubiquitinase, is a partner in the mammalian PRICKLE-interactome. Inhibition of USP9X can arrest PRICKLE-mediated seizures [163]. No studies in humans

Adverse reactions		
	HLA-B*15:02	Stevens-Johnson syndrome and toxic epidermal necrolysis induced by carbamazepine and other aromatic AEDs in patients from Han Chinese and other South Asian ethnic groups [165], [166], [167], [168], [169], [170]- established evidence
	HLA-A*31:01	Increased risk of carbamazepine-induced hypersensitivity reactions in patients of European ancestry and in the Japanese population [172], [173]- established evidence
	T1405 polymorphism of the *CPS1* gene	Increased risk of valproate-induced hyperammonaemia in Caucasian patients [176]
	Val16Ala polymorphism of the *SOD2* gene	Elevated serum level of γ-glutamyltransferase induced by valproate in Japanese patients [179]
	Polymorphic *LEPR* and *ANKK1* genes	Weight gain on valproate in Han Chinese patients [180]
	Variation in *CYP2C9* and *CYP2A6* genes	Risk of toxicity from valproate [181], [182]

### 8.1 Future of pharmacogenomics: new paradigms for testing and translation?

Recent advances in genetic testing are at the heart of current research on the genetic factors influencing drug response and adverse drug reactions in epilepsy. Genetic, and especially genomic, testing is likely to be increasingly used to improve the effectiveness and safety of epilepsy treatments. Precision medicine treatments represent a new incarnation of the age-old physician’s paradigm to consider treatment for each patient holistically: genetics may permit therapy directed at reversing or circumventing the pathophysiological effects of specific gene mutations. Precision medicine could transform clinical care in epilepsy in the form of a new treatment framework and prognostic tools.

A systematic approach to precision medicine in epilepsy should be based on: large cohorts of people with epilepsy with deep genotype-phenotype correlations; standardised functional characterisation of gene mutations which have had frequency and types determined in cases and controls; and the initiation of well-designed innovative clinical trials of targeted treatments identified through functional work. This will require new and more intensive collaborations and integrated research groups that bring together researchers with clinical, genetic, pharmacological and biological expertise.

## Funding

We are grateful for support from the Wellcome Trust (Strategic Award, WT104033AIA), the Epilepsy Society, The Muir Maxwell Trust and European Commission FP7 grant award 279062, ‘EpiPGX’.
